# Using mid-infrared spectroscopy and supervised machine-learning to identify vertebrate blood meals in the malaria vector, *Anopheles arabiensis*

**DOI:** 10.1186/s12936-019-2822-y

**Published:** 2019-05-30

**Authors:** Emmanuel P. Mwanga, Salum A. Mapua, Doreen J. Siria, Halfan S. Ngowo, Francis Nangacha, Joseph Mgando, Francesco Baldini, Mario González Jiménez, Heather M. Ferguson, Klaas Wynne, Prashanth Selvaraj, Simon A. Babayan, Fredros O. Okumu

**Affiliations:** 1 Environmental Health and Ecological Science Thematic Group, Ifakara Health Institute, Morogoro, Tanzania; 2 Institute for Disease Modeling, Bellevue, WA 98005 USA; 3 School of Chemistry, University of Glasgow, Glasgow, G12 8QQ UK; 4 Institute of Biodiversity, Animal Health and Comparative Medicine, University of Glasgow, Glasgow, G12 8QQ UK; 5 School of Public Health, University of Witwatersrand, Johannesburg, South Africa

**Keywords:** Mid-infrared spectroscopy, Supervised machine learning, Malaria, *Anopheles arabiensis*, Mosquito blood meals, Ifakara, Vector surveillance

## Abstract

**Background:**

The propensity of different *Anopheles* mosquitoes to bite humans instead of other vertebrates influences their capacity to transmit pathogens to humans. Unfortunately, determining proportions of mosquitoes that have fed on humans, i.e. Human Blood Index (HBI), currently requires expensive and time-consuming laboratory procedures involving enzyme-linked immunosorbent assays (ELISA) or polymerase chain reactions (PCR). Here, mid-infrared (MIR) spectroscopy and supervised machine learning are used to accurately distinguish between vertebrate blood meals in guts of malaria mosquitoes, without any molecular techniques.

**Methods:**

Laboratory-reared *Anopheles arabiensis* females were fed on humans, chickens, goats or bovines, then held for 6 to 8 h, after which they were killed and preserved in silica. The sample size was 2000 mosquitoes (500 per host species). Five individuals of each host species were enrolled to ensure genotype variability, and 100 mosquitoes fed on each. Dried mosquito abdomens were individually scanned using attenuated total reflection-Fourier transform infrared (ATR-FTIR) spectrometer to obtain high-resolution MIR spectra (4000 cm^−1^ to 400 cm^−1^). The spectral data were cleaned to compensate atmospheric water and CO_2_ interference bands using Bruker-OPUS software, then transferred to Python™ for supervised machine-learning to predict host species. Seven classification algorithms were trained using 90% of the spectra through several combinations of 75–25% data splits. The best performing model was used to predict identities of the remaining 10% validation spectra, which had not been used for model training or testing.

**Results:**

The logistic regression (LR) model achieved the highest accuracy, correctly predicting true vertebrate blood meal sources with overall accuracy of 98.4%. The model correctly identified 96% goat blood meals, 97% of bovine blood meals, 100% of chicken blood meals and 100% of human blood meals. Three percent of bovine blood meals were misclassified as goat, and 2% of goat blood meals misclassified as human.

**Conclusion:**

Mid-infrared spectroscopy coupled with supervised machine learning can accurately identify multiple vertebrate blood meals in malaria vectors, thus potentially enabling rapid assessment of mosquito blood-feeding histories and vectorial capacities. The technique is cost-effective, fast, simple, and requires no reagents other than desiccants. However, scaling it up will require field validation of the findings and boosting relevant technical capacity in affected countries.

## Background

The Global Technical Strategy for Malaria Elimination 2016–2030 [1] recommends that countries should integrate effective surveillance as a core intervention in their malaria policies. As such, the World Health Organization (WHO) recently provided guidelines to support measurements of the most important parasitological and entomological indicators [2]. Effective entomological surveillance requires detailed quantitative understanding of key biological attributes which influence overall potential of vector populations to transmit *Plasmodium* to humans [3]. Such attributes may include the likelihood with which specific *Anopheles* populations bite humans as opposed to the other available vertebrate hosts, i.e. the human blood indices (HBI), defined as proportion of all mosquito blood meals obtained from humans [4, 5]. Other attributes include parasite infection rates, i.e. the proportion of females infected with *Plasmodium* [6], survivorship, i.e. whether the mosquitoes can live long enough to allow complete sporogonic development of *Plasmodium* inside them [7], mosquito susceptibility to insecticides commonly used to control them [8], and the location of mosquito biting, i.e. indoors or outdoors, and how it overlaps in space and time with humans [9–12].

Accurate identification of mosquito blood meal sources is important for understanding host–vector interactions, and provides essential information on transmission dynamics of mosquito-borne diseases [13, 14]. Until recently, blood meals in haematophagous insects were typically identified using immunological assays such as the latex agglutination test [15], precipitin test [16] or enzyme-linked immunosorbent assays (ELISA) [13]. Kent et al. published the first polymerase chain-reaction (PCR) based assay, which addressed many limitations of previous methods and enabled accurate detection of blood meals in field-collected mosquitoes up to several hours post-feeding on cows, dogs, human, pigs, and goats [14]. Lately, other techniques, such as matrix-assisted laser desorption ionization-time of flight mass spectrometry (MALDI-TOF MS) has been applied for mosquito blood meal identification [17–19]. Today, the ELISA [13, 20] and PCR [14] assays are the primary reference methods for measuring HBI in malaria vectors used by most laboratories. Despite generally offering reliable results, these procedures are time-consuming and require repeated supply of reagents making them expensive and unreliable in poorly-resourced settings. Additionally, the ELISA assays are prone to cross-reactivity if laboratory standards are not regularly updated [21].

**Fig. 1:**
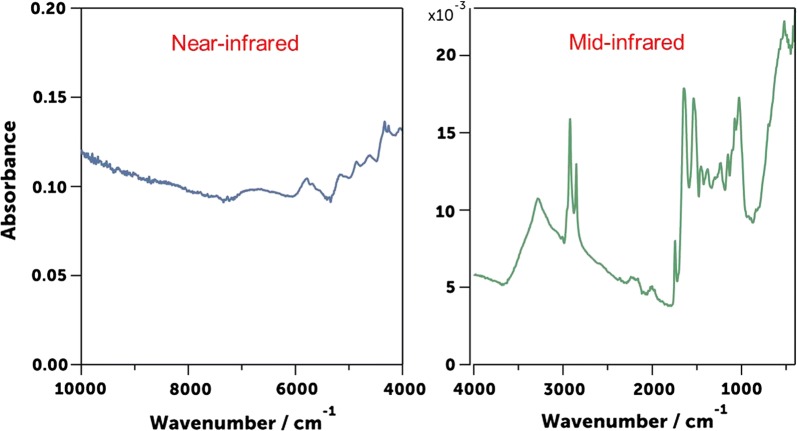
Differences between NIR and MIR spectra obtained from dried mosquito samples collected using ATR-FTIR spectrometer. Compared to near-infrared (NIR), mid-infrared (MIR) allows detection of changes in chemical composition of the samples. Its wavelengths are more sensitive to fundamental vibration of molecular bonds and the different isolated peaks contain information of different chemical components in the mosquito cuticle

Non-molecular techniques such as infrared spectroscopy may offer just as effective but cheaper, quicker, reagent-free and potentially simpler alternatives for assessing key malaria transmission indicators. Indeed, studies have shown that near-infrared (NIR) spectroscopy coupled with chemometrics (mathematical methods of understanding chemical systems) can predict different mosquito ages [22–27], distinguish between mosquito species [22, 28] and even detect presence or absence of pathogens such as *Wolbachia* bacteria, *Plasmodium* and Zika virus in the mosquitoes [29–32]. These successes could be vastly improved by using more effective analytical approaches to process spectral data. Further improvements could potentially be achieved by relying on mid-infrared (MIR) wavelengths (4000 cm^−1^ to 400 cm^−1^), which compared to those of NIR (12,500 cm^−1^ to 400 cm^−1^), Fig. 1 also allow detection of changes in chemical composition of samples, and can clearly show contributions of different chemical bonds of product constituents in separate peaks [33].

This current study investigated the potential of using supervised machine learning algorithms and MIR spectroscopy to accurately distinguish between blood meals of four different vertebrate species within abdomens of the malaria vector, *Anopheles arabiensis.*

## Methods

### Mid-infrared spectrometer

A Bruker ALPHA Fourier-transform infrared (FTIR) spectrometer equipped with a Platinum ATR device was used [34]. The spectrometer had a platinum diamond sampling module with a spectral range of 375–7500 cm^−1^ and maximum spectral resolution between 2 and 0.8 cm^−1^. The infrared optical window was fitted with zinc selenide (ZnSe) to accommodate high humidity conditions.

**Fig. 2:**
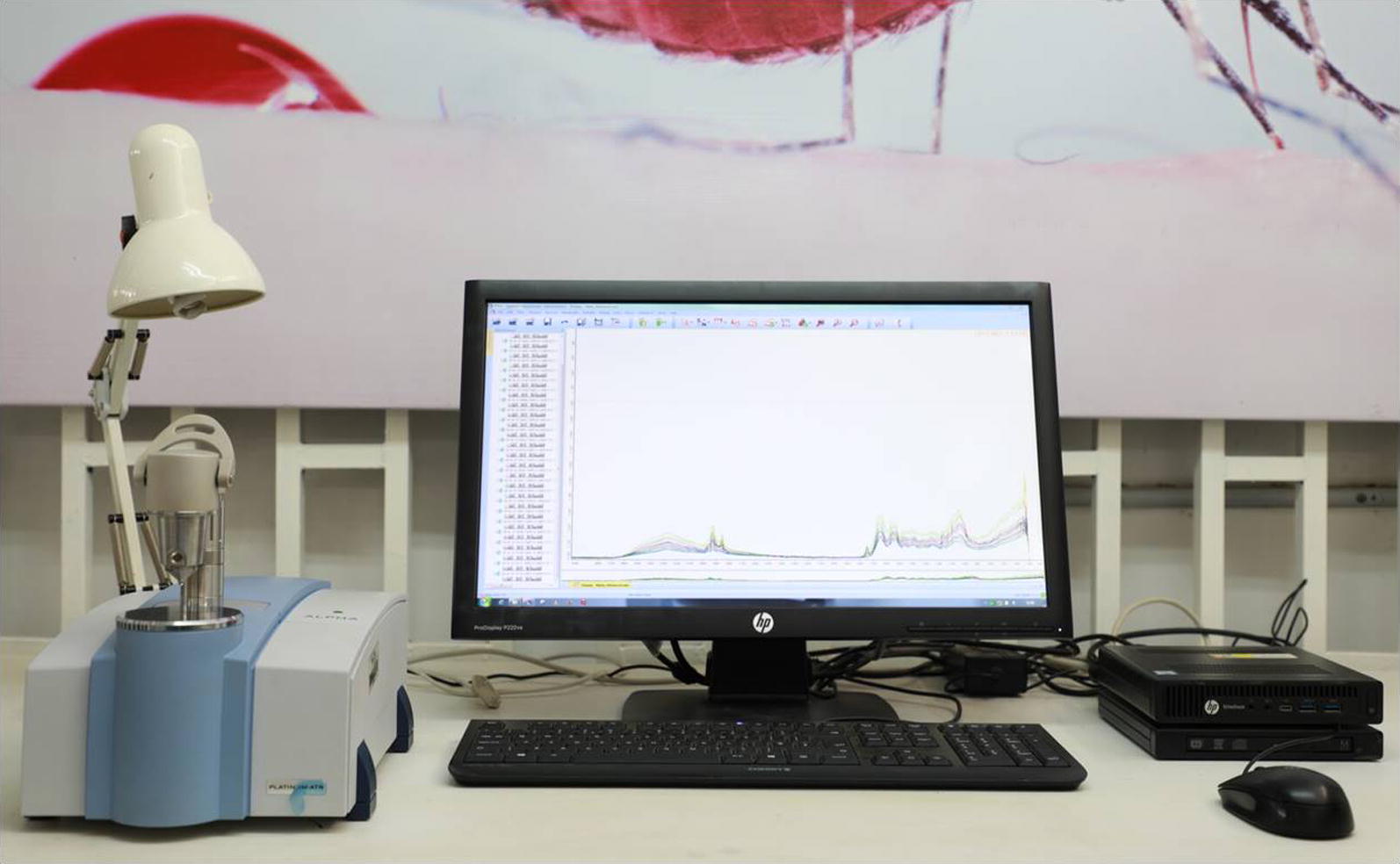
Mid-infrared ALPHA spectrometer with attenuated total reflectance (ATR), and single reflexion diamond platinum crystal, installed at the VectorSphere, Ifakara Health Institute, Tanzania. A control computer is included for the operator

The unit is small (22 cm × 30 cm), highly portable (Fig. 2) and has a permanently aligned interferometer for precise data acquisition [35]. It was installed with an internal validation unit (IVU) with reference standards and programmed to conduct automated instrument tests for operational and performance qualification. Its core is encased in robust metal housing with a lifespan greater than 10 years, and requires minimal maintenance other than replacement of desiccants depending on humidity inside the mid-IR spectrometer [35].

The spectrometer together with the operating computer was installed in the vector biology laboratory, the VectorSphere, at Ifakara Health Institute (IHI), Ifakara, Tanzania (Fig. 2). Proprietary OPUS software version 7.5 [36], licensed to IHI, was also installed to record and process the MIR spectra.

### Mosquitoes

The malaria vector *An. arabiensis* was used in this study because of the natural plasticity of its blood-feeding preferences, and its readiness to feed on non-human hosts when humans are not available [4, 5]. Laboratory-reared females were used. Larvae were reared in plastic basins and fed on Tetramin^®^ fish food (Tetra GmbH, Melle, Germany), while adults were maintained on 10% sugar meals and human arm-feeding for colony maintenance. Adult mosquitoes were maintained at temperature of 27 ± 2 °C, relative humidity (RH) of 80 ± 5% as previously described [37]. Females aged 4–6 days old were used for experiments, all starved for 6 h prior to direct blood feeding as described below.

### Mosquito blood-feeding on different vertebrate hosts

We identified four vertebrate host species widely available and commonly fed upon by *Anopheles* mosquitoes [5, 38] in rural Tanzania. These included: bovine, chicken, goat, and human. For each host species, five individuals were recruited, and 100 female *An. arabiensis* fed upon each one. This way, every host species had 500 blood-fed mosquitoes (100 per individual host/replicate). All humans were all males and recruited from the research team, and other animal hosts (i.e. bovine, chicken, and goat) were bought and were part of the research project. All hosts were restrained and mosquitoes were fed until they were fully engorged. The blood-feeding took place over several days so that the groups consisted of individual mosquitoes from different reproductive batches in the mosquito colony. After blood-feeding, mosquitoes were held for 6 h for digestion to begin and to minimize potential differences associated with extent of blood digestion in the gut [39]. After the holding period, the mosquitoes were killed using chloroform [33] and preserved in micro-centrifuge tubes with silica gel to keep them dry before scanning. Each sample was labelled by date, vertebrate host type, mosquito species, sample ID and age.

### Scanning the preserved mosquitoes

Abdomens were first separated from the heads and thoraxes of the dried mosquitoes. Since the study was primarily focused on gut content, the heads and thoraces were discarded and instead only the abdomens were scanned. The mosquito specimen (abdomen) was placed at the center of the MIR crystal plate, and supported by the spectrometer arm (the anvil). MIR spectra were captured (spectral range 4000–400 cm^−1^), with spectral resolution set at 5 cm^−1^. Each individual specimen was scanned 32 times in 30 s, and resulting spectra averaged to obtain a single representative spectrum as previously described [33]. The spectra were recorded in absorbance units and stored using Bruker OPUS software [36].

### Pre-processing of the MIR spectra

The OPUS software [36] was used to clean and compensate spectra with water vapor absorption bands (intense bands centered around 2340 cm^−1^, 3600 and 550 cm^−1^ wavenumbers) and carbon dioxide (CO_2_) interference bands (4000–3400 cm^−1^ and 2200–1300 cm^−1^) as previously described [33]. The cleaned spectral data were converted from the Bruker OPUS format to text files in Python™. Further spectral cleaning was done by discarding spectra with low intensities (below 0.11 absorbance units; mostly between 400 and 500 cm^−1^) and spectra with no features (flat spectra) [33]. The final cleaned spectra matrix was saved in comma separated values (CSV) format for further analysis.

### Data training, validation, and prediction of blood meal sources

**Fig. 3:**
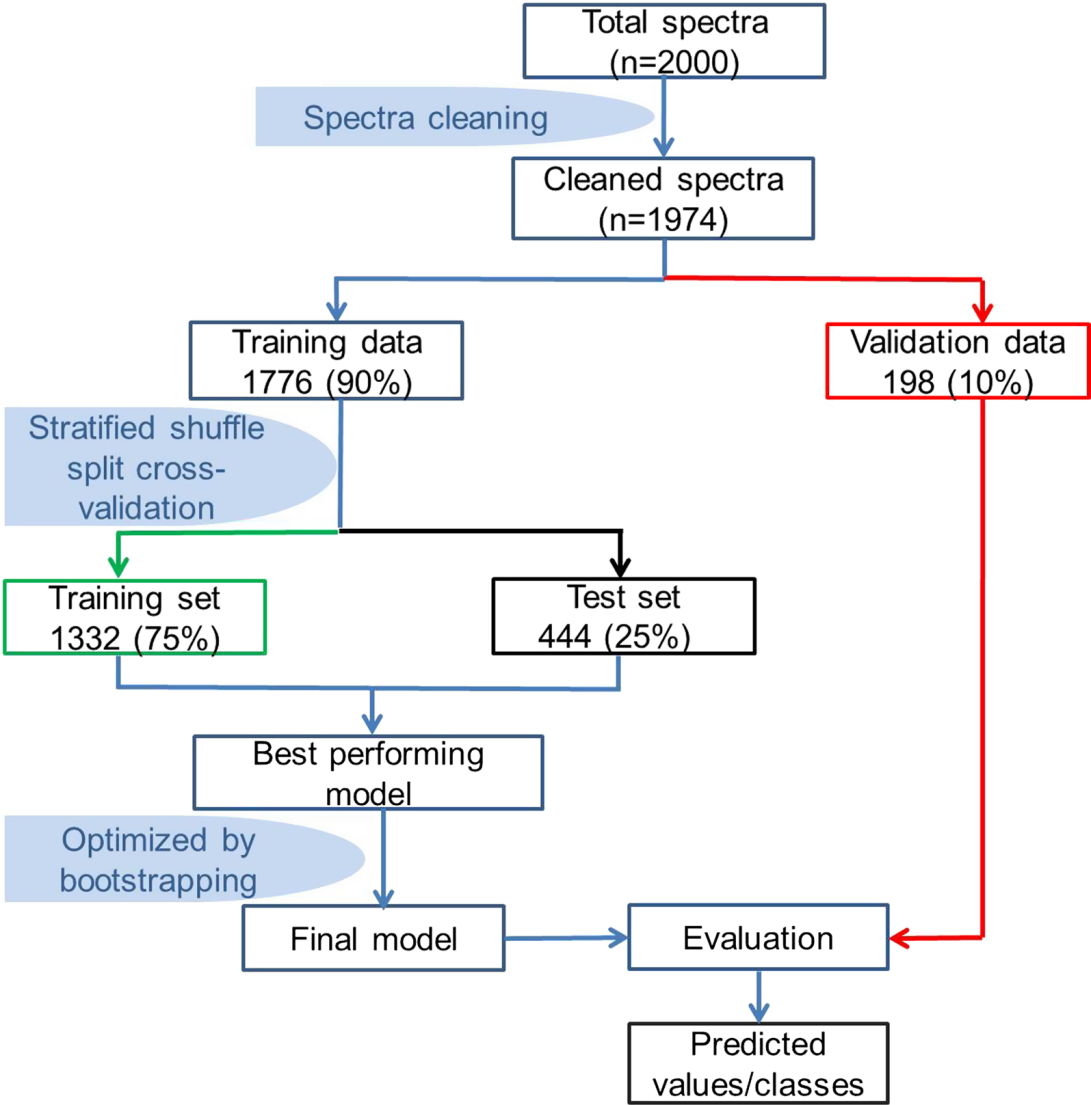
Schematic illustration of the processes of data splitting, model training, cross-validation and evaluation of performance of final model

The MIR spectra were analyzed in the Python™ programming language with the *scikit*-*learn* library [40]. Supervised machine learning techniques were used to train models on known and predicted classes for the validation set, based on a multi-class classification strategy [40]. Models were trained and cross-validated using the strategy as illustrated in Fig. 3. First, the whole dataset was partitioned into training (90% of the data) and validation datasets (10%), ensuring proportional representation of the different host types and individuals (Fig. 3). The validation set was split from the whole dataset before training process such that the 10% subset was neither used for training nor for selection of the trained models. Instead, it was preserved for evaluating accuracies of the final model. Once the training dataset was separated, it was itself subjected to multiple rounds of randomly-stratified splits into training sets (75%) and test sets (25%) as illustrated in Fig. 3, to achieve rigorous classification of the different blood meal types, this time involving only the training dataset. The spectra data were used to classify blood meals into one of four host species classes.

To achieve this, classification algorithms were used to learn patterns from matrices of features which represent different blood meal sources. Seven different algorithms were tested using default settings on the training set. The candidate algorithms included: k-nearest neighbours classifier (KNN), logistic regression (LR), support vector machine classifier (SVM), naïve Bayes (NB), random forest classifier (RF), XGBoost classifier (XGB), Multilayer perceptron (MLP) [40]. Prediction scores were presented in terms of percentage accuracy.

Finally, the best performing model of the seven above, i.e. model with highest accuracy (percentage of times a blood meal was correctly classified to the right host species) and precision (variability between actual estimates), was selected. The model was optimized by fitting 100 bootstrapping regressions, and bagged to increase prediction performance. Performance of this final model was evaluated using the validation set.

## Results

Of the 2000 individual spectra collected, 26 randomly distributed were discarded during data cleaning because of sub-standard quality. Of the remaining 1974 spectra, 1776 were used in training and testing of supervised machine learning models and 198 were used for final validation of the final model.

**Fig. 4:**
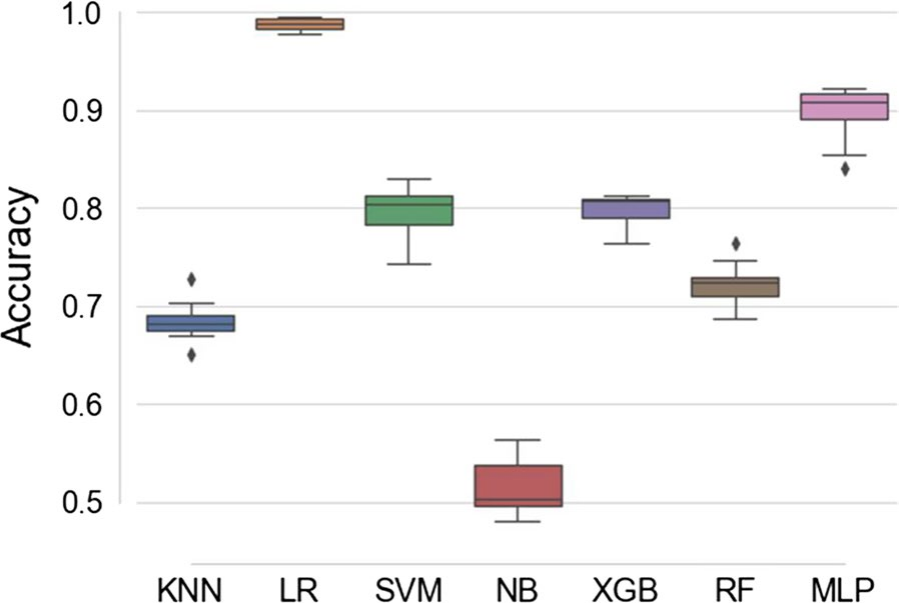
Prediction accuracies for different classification algorithms. Models tested include k-nearest neighbours (KNN), logistic regression (LR), support vector machines (SVM), naïve Bayes (NB), XGBoost (XGB), random forest (RF), Multilayer perceptron (MLP). Based on prediction accuracy and precision achieved, the best performing model was LR

**Fig. 5:**
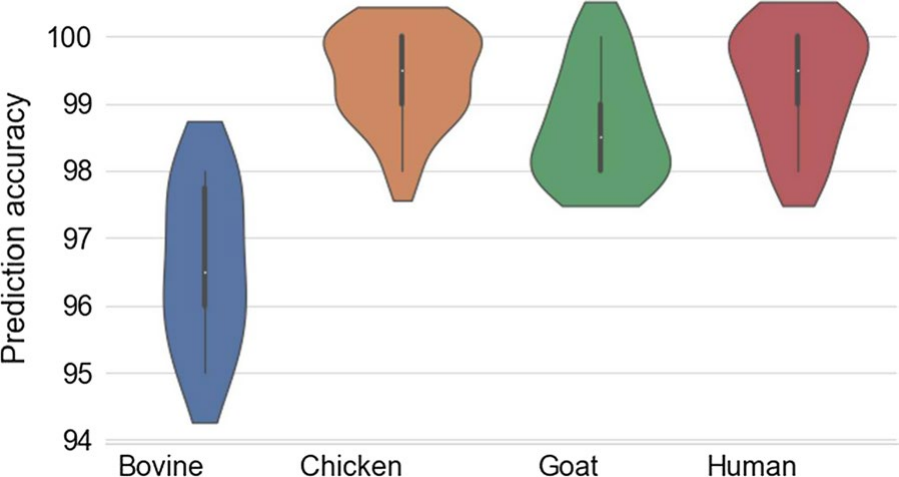
Prediction accuracies obtained by the final logistic regression (LR) model for different vertebrate blood meal sources. Distribution around the prediction accuracy indicates standard deviation in the 100 bootstrapped models and is used to assess model precision

**Fig. 6:**
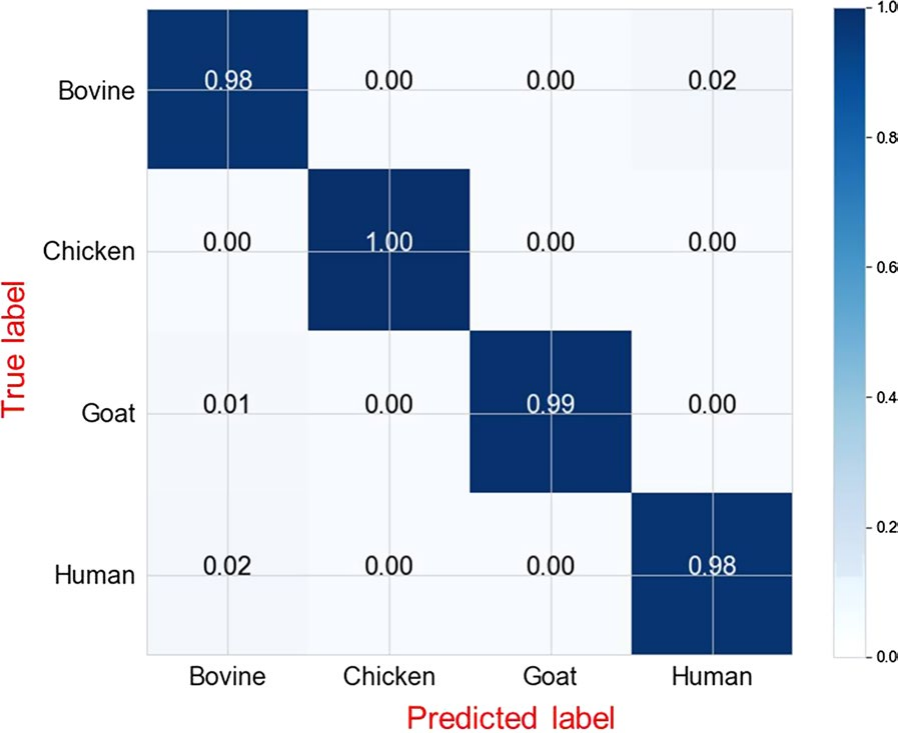
Normalized confusion matrix for the trained model (training set = 1332 spectra; test set = 444 spectra; total spectra = 1776). Each row represents instances in actual class (true label), while each column represents instances in predicted class (predicted label). From the top left to bottom right, the blue line highlights final prediction accuracies in each class

Of the seven classification algorithms tested, logistic regression (LR) was identified as the best approach since it outperformed the other six classifiers in identifying mosquito blood meal sources of laboratory-reared *An. arabiensis* (Fig. 4). After additional optimization by bootstrapping, LR successfully predicted mosquito blood meal sources by correctly identifying the source of host blood meals more than 90% of the time. A total of 100 bootstrapped models were fitted, which when aggregated predicted mosquito blood meals with an overall accuracy of 98.6%, Fig. 5). Average accuracies by class were 98% for bovine and human blood, 99% for goat blood, and 100% for chicken blood (Fig. 6).

**Fig. 7:**
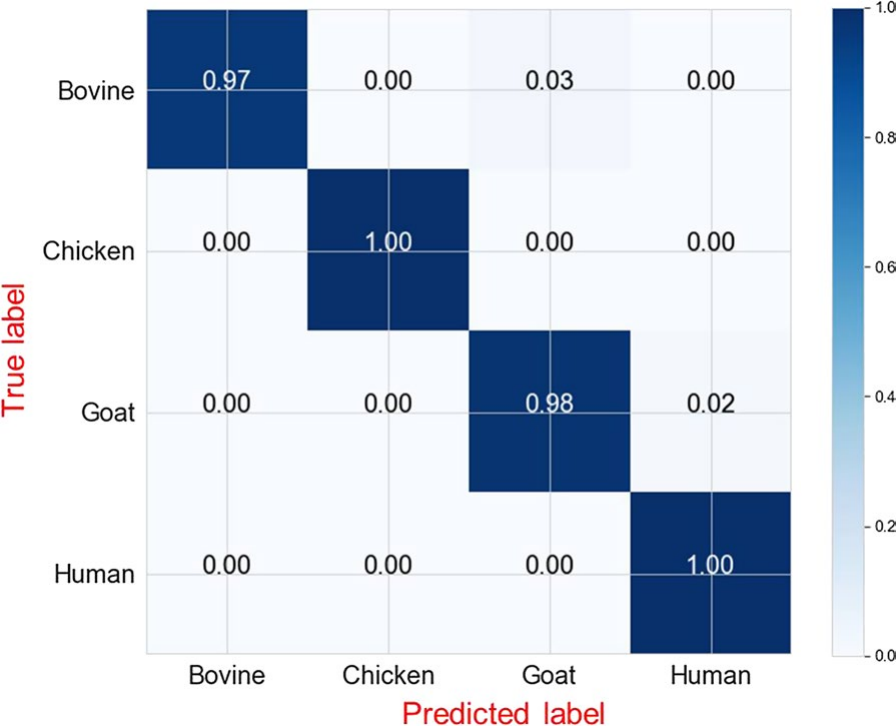
Normalized confusion matrix for final model evaluation (training data = 1776 spectra; validation data = 198 spectra; total spectra = 1974). Each row represents instances in actual class (true label), while each column represents instances in predicted class (predicted label). From the top left to bottom right, the blue line highlights final prediction accuracies in each class

In the final validation on held-out 198 previously unseen spectra, the optimized model predicted correct identities of blood meals in this new dataset by 98.4% overall accuracy (96% for goat blood, to 97% for bovine blood and 100% for chicken and human blood) (Fig. 7). Three-percent of bovine blood samples were misclassified as goat blood and 2% of goat blood misclassified as human blood (Fig. 7).

## Discussion

This work has demonstrated that mid-infrared spectroscopy (MIR) coupled with supervised machine learning can accurately distinguish between mosquito blood meals originating from common vertebrate hosts, i.e. bovine, chicken, goat and human, without requiring molecular techniques. It is also represents the first evaluation of an infrared-spectroscopy approach for blood meal analysis, and application of machine learning to analyze mosquito blood feeding histories spectral data. The experiments were done using laboratory-reared *An. arabiensis* mosquitoes but with known blood hosts replicated across five individuals for each host type. *An. arabiensis* was selected for these experiments because of its natural plasticity of host preferences [4, 5], but similar analyses would apply for all other mosquito species.

This work builds upon a series of studies using NIR spectroscopy, most of which have already demonstrated the potential of these technologies for analysis of several important mosquito traits. Examples include prediction of different mosquito ages [22–27], use of NIR spectroscopy and chemometrics to distinguish between two malaria vector species, *Anopheles gambiae* sensu stricto and *An. arabiensis* [22, 28], and detection of pathogens such as Zika virus [30], malaria parasites [31, 32] and the symbiont, *Wolbachia* [29, 30].

Until now a key challenge of this approach has been the lack of high-capacity statistical tools to handle the massive quantities of spectral data generated from the procedures, and also lack of field validation of many of these approaches. Both these challenges are now on the verge of being addressed by multiple research groups. As recently described by González-Jiménez et al. [33], this current study also applied MIR as opposed to the NIR wavelengths previously used. MIR extends from 4000 to 400 cm^−1^, and is between the far-infrared (FIR) region (400 cm^−1^ to 10 cm^−1^) and NIR region (12,500 cm^−1^ to 400 cm^−1^) [41]. As shown in Fig. 1, it records spectra with greater information content compared to NIR. Moreover, key features of MIR spectra such as number of infrared absorption bands and their intensities combined with the advantage of robust instrumentation available for MIR such as used here [34]. The mosquito abdomen was squashed during the scanning so blood meal was present at the surface of the specimen. Different isolated peaks in MIR contain information of different chemical components in the mosquito cuticle, at it appears proteins and lipids may be responsible for the difference in spectra for different vertebrate as also observed in MALDI-TOF MS approach [17–19]. The MIR-based approach has great potential for accuracy and scalability. In this study, only mosquito abdomen was used but also other parts (head and thorax) could be used to distinguish between species and age.

To process the MIR data, this study deployed multiple supervised machine learning algorithms before selecting the most accurate and precise candidate for final analysis. Machine learning is increasingly applied in medical and public health industry [33, 42–48], and will likely become dominant in disease predictions and surveillance [42, 43]. For example, it has been used to solve problems in genomic medicine [44, 45] and to predict responses to antiretroviral treatment, in one case with ~ 78% accuracy [46]. Nearly 10 years ago, the approaches were already being used to predict diabetes and pre-diabetes outcomes with greater than 80% accuracy [47]. Recently, Chen et al. proposed a faster neural network approach based on multimodal disease risk prediction using data from health care facilities [48]. This approach reached 94% accuracy in evaluating risk of cerebral infarction disease.

In this current work, logistic regression (LR) models were found to be the best performing for quantifying variations of MIR spectral information on mosquito blood meal sources. Optimization of the LR model with 100 bootstrapped realizations of the dataset led to very high prediction accuracy of 98.8%, achieving perfect score in a few instances (Fig. 4). The selected model remained very highly accurate, exceeding 98% even when challenged with a new dataset not previously seen by the model.

The technique has shown to be highly effective, achieving accuracies previously achieved by ELISA [13] and PCR. However, the samples here were prepared just 6 to 8 h after blood-feeding on known animal bloods, so future studies should consider using different digestion stages as this may influence accuracy [14, 39, 49]. The technique will need to be evaluated whether it can detect and distinguish blood feeding histories of malaria mosquitoes for more than 6 h as well as mixed blood meals. In this study, there was no uniformity between the individuals of the same host species in terms of attributes such as sex, age, weight and health status. Mosquitoes selected in this study were fully engorged; it is still unknown whether the technique will also detect mosquitoes with partial blood meals. Additional advantages of this technique over direct ELISA include the fact that it is time-saving in both sample preparation and analysis, and has reduced cross-reactivity. Despite the fact that PCR is even more sensitive than ELISA and has low risk of cross reactivity, they still consume time and require skilled expertise in deoxyribonucleic acid (DNA) extraction [14]. Lastly, all mosquito samples used in this study were blood-fed on known hosts in a controlled environment. Future work should therefore validate these findings using field collections, in which case PCR assays would be the best standard reference [14].

The costs are also significantly lower, and particularly since no reagents are required other than desiccants. ELISA systems currently cost approximately 13,044 USD, while the cost for MIR spectrometer we used (Bruker ALPHA Fourier-transform infrared (FTIR) spectrometer equipped with a platinum ATR) was approximately 29,000 USD, including shipment and installation, and the costs were incurred only at the initial purchase of the machine. Unlike the MIR spectrometry, ELISA systems require additional reagents, such that the cost per sample can be between 1 and 1.5 USD. MIR is more cost-effective as it does not require repeated reagents for sample processing. This means that in an active laboratory, it would take just 1 year for the overall financial investment in ELISA systems to exceed the costs of MIR systems, and thereafter the costs per sample would continue to reduce. The turnaround time between sample preparation and results for ELISA is approximately 2 days for every 100 samples (including sample preparation, processing and results reading). On the other hand, once the MIR system has been calibrated and established, users can scan 300 or more samples in a day, each sample taking approximately 1 min to scan. The scale of use also suggests that this tool could greatly improve district-wide or nation-wide vector surveillance efforts.

## Conclusion

In conclusion, mid-infrared spectroscopy coupled with supervised machine learning can accurately identify multiple vertebrate blood meals in malaria vectors, thus enabling rapid assessment of mosquito blood-feeding histories and vectorial capacities. The technique is cost-effective, fast, simple and requires no reagents other than desiccants. All the analyses were done in open source software, except for data extraction done using the proprietary Bruker OPUS software, but which can also be done with available open source scripts. Nonetheless, scaling up this approach will require field validation of the findings and specific training to improve technical capacity in affected countries. Once validated, this approach could potentially replace current molecular techniques for blood meal analysis (i.e. PCR and ELISA).

## Data Availability

All data for this study will be available upon request.

## Abbreviations

HBI: Human Blood Index
ELISA: enzyme-linked immunosorbent assay
PCR: polymerase chain reaction
MIR: mid-infrared
ATR-FTIR: attenuated total reflectance-Fourier transform infrared spectrometer
MALDI-TOFMS: matrix-assisted laser desorption ionization-time of flight mass spectrometry
NIR: near-infrared
ATR: attenuated total reflectance
FTIR: Fourier transform infrared spectrometer
KNN: k-nearest neighbours
LR: logistic regression
NB: naïve Bayes
RF: random forest
XGB: gradient boosting
MLP: Multilayer perceptron
